# In vitro anti-malarial efficacy of chalcones: cytotoxicity profile, mechanism of action and their effect on erythrocytes

**DOI:** 10.1186/s12936-019-3060-z

**Published:** 2019-12-16

**Authors:** Shweta Sinha, Daniela I. Batovska, Bikash Medhi, B. D. Radotra, Ashish Bhalla, Nadezhda Markova, Rakesh Sehgal

**Affiliations:** 1 Department of Medical Parasitology, Post Graduate Institute of Medical Education and Research, Chandigarh, 160012 India; 2 Institute of Organic Chemistry with Centre of Phytochemistry, Bulgarian Academy of Sciences, Sofia, Bulgaria; 3 Department of Pharmacology, Post Graduate Institute of Medical Education and Research, Chandigarh, India; 4 Department of Histopathology, Post Graduate Institute of Medical Education and Research, Chandigarh, India; 5 Department of Internal Medicine, Post Graduate Institute of Medical Education and Research, Chandigarh, India

**Keywords:** Malaria, *Plasmodium falciparum*, Chalcones, In vitro, Haemozoin

## Abstract

**Background:**

Malaria extensively leads to mortality and morbidity in endemic regions, and the emergence of drug resistant parasites is alarming. Plant derived synthetic pharmaceutical compounds are found to be a foremost research to obtain diverse range of potent leads. Amongst them, the chalcone scaffold is a functional template for drug discovery. The present study involves synthesis of ten chalcones with various substitution pattern in rings A and B and assessment of their anti-malarial efficacy against chloroquine sensitive and chloroquine resistant strains as well as of their cytotoxicity and effect on haemozoin production.

**Methods:**

The chalcones were synthesized by Claisen-Schmidt condensation between equimolar quantities of substituted acetophenones and aryl benzaldehydes (or indole-3-carboxaldehyde) and were screened for anti-malarial activity by WHO Mark III schizont maturation inhibition assay. The cytotoxicity profile of a HeLa cell line was evaluated through MTT viability assay and the selectivity index (SI) was calculated. Haemozoin inhibition assay was performed to illustrate mode of action on a *Plasmodium falciparum* strain.

**Results:**

The IC_50_ values of all compounds were in the range 0.10–0.40 μg/mL for MRC-2 (a chloroquine sensitive strain) and 0.14–0.55 μg/mL for RKL-9 (a chloroquine resistant strain) of *P. falciparum*. All the chalcones showed low cellular toxicity with minimal haemolysis. The statistically significant reduction (p < 0.05) in the haemozoin production suggests a similar mechanism than that of chloroquine.

**Conclusions:**

Out of ten chalcones, number **7** was found to be a lead compound with the highest potency (IC_50_ = 0.11 µg/mL), as compared to licochalcone (IC_50_ = 1.43 µg/mL) and with high selectivity index of 85.05.

## Background

Malaria control programmes are threatened due to a rapid expansion of resistance to distinct anti-malarial drugs. At present, 219 million cases are reported at a global scale, mostly in children under 5 years of age [1]. Out of the five species that cause human malaria, *Plasmodium falciparum* and *Plasmodium vivax,* are associated with life-threatening complications. There is confirmed resistance of both species against most of currently available anti-malarials. To combat drug resistant *Plasmodium*, artemisinin and its derivatives have been widely implicated all over in endemic regions, but appearance of artemisinin resistance, first in Cambodia in 2007 [2] and later its rapid spread to the south-east Asian region [3–7] has threatened all the previous success incurred by malaria control strategies.

**Fig. 1:**
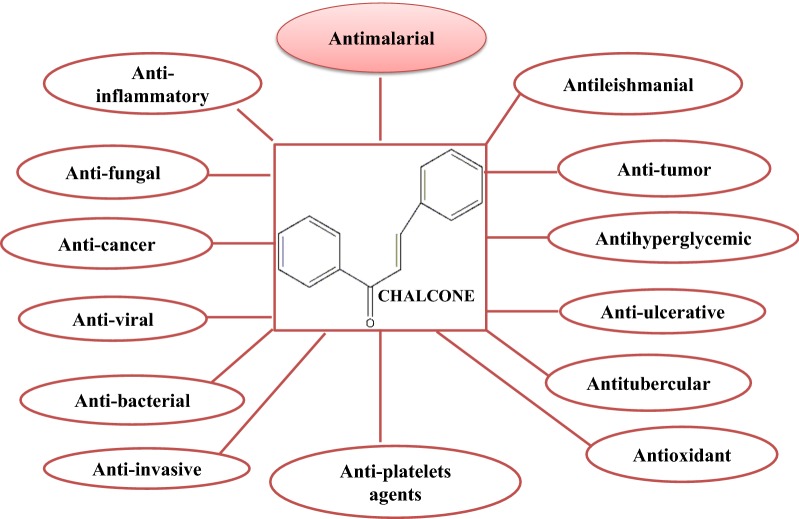
Diverse pharmacological activity of chalcones

Chalcones (1,3-diaryl-2-propen-1-ones) are basically plant secondary metabolites related to flavonoid family and are also crucial precursors of distinctive flavonoids and isoflavonoids [8]. They have been extensively studied due to their diverse pharmacological actions [9, 10], including anti-malarial activity (Fig. 1) [11, 12].

**Fig. 2:**
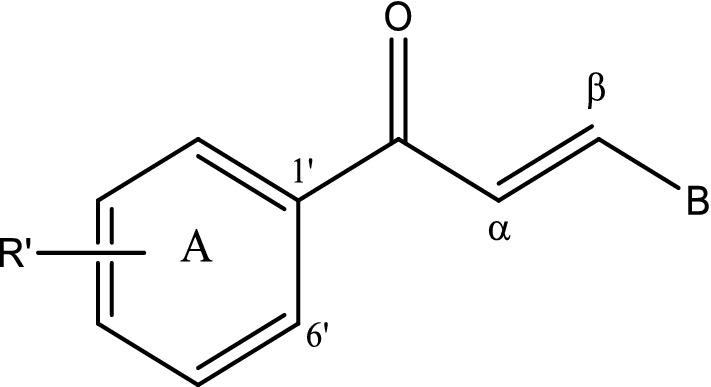
Basic structure of chalcones

Moreover, chalcones can be simply synthesized by the cost-efficient Claisen-Schmidt condensation between variously substituted benzaldehydes and acetophenones [13] thus, providing an array of distinctive potential analogues with potent pharmacological effects [14]. Anti-malarial activity of such chalcones is mostly attributed to the specificity of the substitution pattern, and hydrophobicity and size of ring B (Fig. 2) [15]. The anti-malarial property of chalcone was first reported after an in vitro evaluation of an oxygenated chalcone, “licochalcone A” exclusively obtained from Chinese licorice, as an anti-malarial agent against chloroquine sensitive and chloroquine resistant *Plasmodium* strains [16]. Further, many more potential analogues of licochalcone A with different substitution pattern have been reported for substantial anti-malarial activity [17]. The simple structure and unambiguous synthesis of chalcones have fascinated the consideration of many chemists to find and expand distinct analogues of this unusual scaffold for various infectious diseases including malaria. For more than a decade, a panel of alkoxylated, prenylated, hydroxylated, quinolinated, oxygenated chalcones derived from either syntheses or natural sources have been assessed for antiplasmodial activity with promising outcomes [17, 18]. Although several mechanisms have been postulated for various chalcones [15, 19–22], the exact mode of action still remains unclear. Besides, these chalcones are mostly supposed to show their anti-malarial activity through preventing host haemoglobin degradation by acting against malarial cysteine protease [23]. Molecular modelling research illustrated the linear and planar structure of chalcones, which enables them to fit appropriately within the active site of *Plasmodium* and *Trypanosoma* cysteine proteases suggesting a promising target for its action [23]. The present study describes synthesis of ten chalcones with different substitution pattern in rings A and B and assessment of their anti-malarial efficacy against chloroquine sensitive and chloroquine resistant strains as well as of their cytotoxicity and mode of action.

## Methods

### Chemicals and reagents

The chalcones were synthesized at the Institute of Organic Chemistry with Centre of Phytochemistry, Bulgarian Academy of Sciences, Sofia, Bulgaria. Chloroquine phosphate, quinine hydrochloride, glutamine, sodium bicarbonate, and β-haematin were purchased from Sigma Aldrich while artemisinin was from IPCA. The study was approved by Institute Ethics Committee Project No. NK/1265/Ph.D/23991 at Post Graduate Institute of Medical Education and Research, Chandigarh, for maintenance of *P. falciparum* strains in human erythrocytes and AB^+ve^ human serum.

### Chemistry

The chalcones were synthesized by Claisen-Schmidt condensation between equimolar quantities of substituted acetophenones and aryl aldehydes (or indole-3-carboxaldehyde) [24, 25]. The progress of the reactions was monitored by thin-layer chromatography on silica gel plates. The condensation step was carried out over 6 h to 36 h. After purification by either column chromatography on silica gel or recrystallization from methanol, all corresponding chalcones were obtained in yields over 90%.

Stock solutions of chloroquine phosphate, quinine hydrochloride, artemisinin and each chalcone were prepared by dissolving each compound in DMSO to achieve concentration of 1.00 mg/mL. The DMSO amount in diluted concentrations (1%) had negligible effect on the parasite growth. DMSO was used as negative control.

### In vitro anti-malarial activity

#### Parasites and culture

Two *P. falciparum* strains, MRC-2 (sensitive to chloroquine) and RKL-9 (resistant to chloroquine), obtained from National Institute of Malaria Research (NIMR), New Delhi, India, were used in this study. These strains were perpetuated in vitro in continuous culture according to the method of Trager and Jensen [26] with slight modifications. Briefly, both sensitive and resistant strains of *P. falciparum* were maintained in A+ erythrocytes in RPMI-1640 medium (having glutamine, but without any sodium bicarbonate) comprising 1.00 g of dextrose, 5.94 g of HEPES buffer, 40.00 mg of gentamycin. Additionally supplemented with 5% sodium bicarbonate and 10% (v/v) inactivated human AB+ serum then incubated in gas mixture of 5% CO2, 5% O2 and 90% N_2_ at 37 °C. Parasitized erythrocytes at initial 5% haematocrit were suspended in above mentioned culture medium and parasitaemia was regularly checked to maintain level between 2 and 4% with further sub-culturing for parasitaemia beyond 5%. Growth and multiplication of parasite was monitored by microscopy using Giemsa-stained slides.

#### Synchronization

To obtain ring stages of the parasite, the cultures were synchronized using d-sorbitol [27]. The cultures, with majority of ring stages, were treated with equal volume of aqueous 5% d-sorbitol for 5 min and then after centrifugation pellet were suspended in complete medium and fresh erythrocytes synchronized culture with 1% parasitaemia and 5% haematocrit were used for compound concentration response assay.

#### Compound concentration response assay

The concentration of each test compound needed to hinder multiplication of parasites by 50% (IC_50_) against *P. falciparum* strains were obtained through concentration response assay performed in 96-well sterile tissue culture plates. Synchronized parasite cultures were applied to different doses of each compound. Dilutions were performed in gentamycin-free culture medium, and incubated at 37 °C having gaseous mixture (5% CO_2_, 5% O_2_ and 90% N_2_) supply for 24 h. The results were expressed as IC_50_ values computed from HN-NonLin Regression analysis [28], as well as mean percentage inhibition ± standard error examined by thick smear Giemsa stained slides [29, 30].

$$\begin{aligned}& \% {\text{Parasite}}\;{\text{Inhibition}}\;\left[ {29,\;30} \right] \\&\quad= 100 - \left[ {\left( {{\text{Total}}\;{\text{number}}\;{\text{of}}\;{\text{Schizonts}}\;{\text{in}}\;{\text{test}}\;{\text{wells}}} \right)/}\right.\\&\qquad\left.{\left( {{\text{Total}}\;{\text{number}}\;{\text{of}}\;{\text{Schizonts}}\;{\text{in}}\;{\text{control}}\;{\text{well}}} \right)*100} \right] \end{aligned}$$

#### Resistance index (RI)

The degree of resistance was determined by comparing the activity of chalcones on the chloroquine sensitive and chloroquine resistant strains of *P. falciparum* using the following formula [31]:

$$\begin{aligned} {\text{RI }}& = {\text{IC}}_{50} \;{\text{Choroquine}}\;{\text{resistant}}\;{\text{strain}}/\\&\quad{\text{IC}}_{50} \;{\text{Chloroquine}}\;{\text{sensitive}}\;{\text{strain}} \end{aligned}$$

### Cytotoxicity assay and evaluation of selective index

Cytotoxicity of the compounds on mammalian cells were accomplished employing HeLa cell line (NCCS, Pune) cultured in DMEM supplemented with 10% FBS by the MTT (3-(4,5-dimethylthiazol-2-yl)-2,5-diphenyltetrazolium bromide) microenzymatic method with certain modifications [32]. Briefly, cells (10^4^ cells/200 μL/well) were seeded into 96-well flat-bottom sterile tissue culture plates in complete medium. After 24 h of seeding, the test compounds at different dilutions were added and kept for another 24 h in a humidified chamber with 5% CO_2_ at 37 °C. Twenty microlitres of MTT (5.00 mg/mL in 1XPBS) stock solution were pipetted into each well, mixed and incubated for at least 3–4 h. After incubation, the plates were centrifuged at 1500 rpm for 5 min. The supernatant was disposed cautiously and 100 μL of DMSO were added to each well to lyse the cell and dissolve the insoluble purple formazan product into a coloured solution. Absorbance was taken at 570 nm to determine formazan formation as a measurement of cell viability. Experiments were performed in triplicate. The 50% cytotoxic concentration (CC_50_) was assesses by analysis of dose–response curves. Selectivity Index (SI) was calculated as [31]:

$${\text{SI }} = {\text{CC}}_{ 50} /{\text{IC}}_{ 50}$$

### Haemolysis assay

Haemolytic effect of all chalcones and standard anti-malarial drugs, chloroquine, quinine and artemisinin, was examined by incubating normal erythrocytes with all above mentioned compounds in phosphate-buffered saline (PBS), respectively. Briefly, fresh erythrocytes were centrifuged for 5 min at 1600 rpm for at least thrice in PBS and then the remaining pellet was re-suspended in PBS at 2% hematocrit. One hundred microlitres of this suspended pellet was added to 96-well sterile culture plate having test compounds at different desired concentrations. PBS alone (for baseline values) and 0.4% Triton X-100 in PBS (for 100% haemolysis) were employed as controls. After keeping at 37 °C for 3 h, the test samples were centrifuged and the supernatant was used for determination of the haemolytic activity quantified in terms of haemoglobin release as monitored spectrophotometrically by taking absorbance at 415 nm [33]. The experiment was done in triplicate and the mean ± SD was calculated [33, 34].

$$\begin{aligned}& \% \;{\text{Haemolysis }} = [\left( {{\text{Absorbance}}\;{\text{of}}\;{\text{sample}}_{{415\;{\text{nm}}}}}\right.\\&\quad\left.{ - {\text{Absorbance}}\;{\text{of}}\;{\text{blank}}\;{\text{sample}}_{{415\;{\text{nm}}}} } \right)/\\&\quad{\text{Absorbance}}\;{\text{of}}\;{\text{ positive}}\;{\text{control}}_{{415\;{\text{nm}}}} ]*100 \end{aligned}$$

### Haemozoin inhibition assay

The haemozoin (β-haematin) inhibition by distinct drugs in *P. falciparum* cultures was assessed employing drug concentrations in the proximity of IC_50_ concentrations after completion of 48 h [35]. Briefly, the test cultures were centrifuged for 5–10 min at 1300 rpm to dispose of the culture medium. Infected erythrocyte pellet (mingled of β-haematin and erythrocyte membrane) were exposed to 0.01% saponin lysis for 10 min at 25 °C to lyse erythrocyte to release parasites. These released parasites were further washed three times with PBS, re-suspended in 2.5% sodium dodecyl sulfate buffer solution (SDS in PBS) and subjected to spin at 20,000 g for 1 h. The supernatant was disposed and the insoluble haemozoin pellet was washed in 2.5% SDS in PBS and then dissolved in 20 mM NaOH. The haemozoin content was quantified by taking the absorbance at 400 nm and using a standard curve prepared from β-haematin. The amount of haemozoin formed in relation to control was calculated. All assays were performed in triplicate.

### Statistical analysis

Data were presented as mean ± SD. IBM SPSS Statistics version 21.0 was used for data analysis. p < 0.05 was taken as level of significance. Means were compared using one-way analysis of variance (ANOVA) followed by post hoc, Bonferroni multiple comparison test.

## Results

### Chemistry

The structures of the synthesized chalcones are represented in Table 1.

**Table 1:** Structure of the synthesized chalcones 1–10

Chalcone	R’	B
1	2′,4′,6′-Trimethoxy-	3,4-Dimethoxyphenyl-
2	2′,5′-Dimethoxy-	4-Methoxyphenyl-
3	2′,5′-Dimethoxy-	3,4-Methylenedioxyphenyl-
4	3′,4′,5′-Trimethoxy-	4-Fluorophenyl-
5	3′,4′,5′-Trimethoxy-	4-Dimethylaminophenyl-
6	3′,4′,5′-Trimethoxy-	4-Methoxyphenyl-
7	3′,4′,5′-Trimethoxy-	3,4-Dimethoxyphenyl-
8	3′,4′,5′-Trimethoxy-	3,4-Methylenedioxyphenyl-
9	4′-Chloro-	1H-Indole-2-yl-
10	4′-Iodo-	1H-Indole-2-yl-

### Anti-malarial activity

**Fig. 3:**
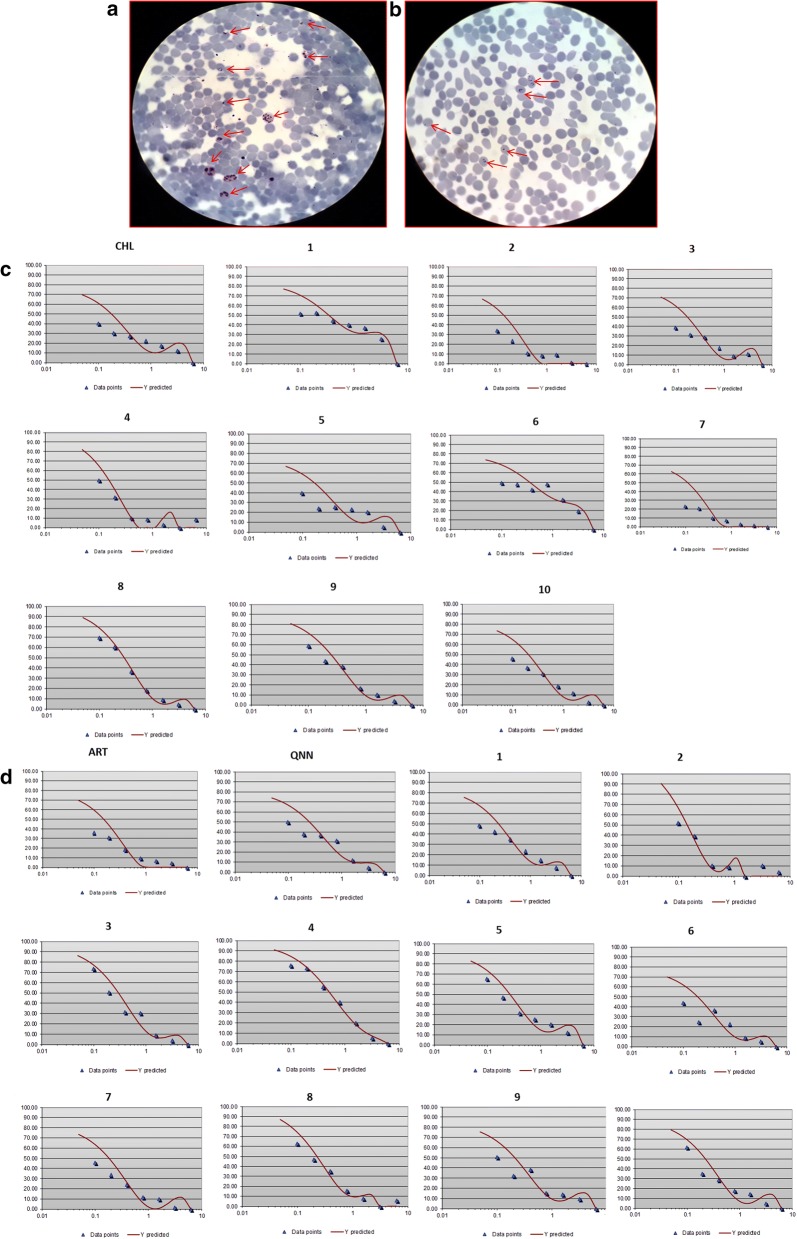
In vitro anti-malarial activity of chalcones on *P. falciparum*. **a** Unsynchronized culture of *P. falciparum* containing different stages of their life cycle; merozoites, early trophozoites (early ring stage), late trophozoites (late ring stage), schizonts, invading merozoites observed from Giemsa-stained slide under 1000× magnification. **b** Synchronized culture containing only ring stages of *P. falciparum* after treatment with 5% D-sorbitol observed from Giemsa-stained slide under 1000X magnification. **c** Dose–response curves (y-axis represents; % parasite matured into schizonts and x-axis represents; log_10_ concentration) of chloroquine sensitive *P. falciparum* strain (MRC-2) to different concentration of chalcones number 1,2, 3, 4, 5, 6, 7, 8, 9 and 10 and chloroquine (CHL). **d** Dose–response curves (y-axis represents; % parasite matured into schizonts and x-axis represents; log_10_ Concentration) of chloroquine resistant *P. falciparum* strain (RKL-9) to different concentration of chalcones number 1, 2, 3, 4, 5, 6, 7, 8, 9 and 10, quinine hydrochloride (QNN) and artemisinin (ART)

The chloroquine sensitive (MRC-2) and chloroquine resistant (RKL-9) strains of *P. falciparum* were cultured in vitro under sufficient gaseous mixture in RPMI1640 medium and the culture was synchronized by treating with 5% d-sorbitol to acquire mainly ring stage *Plasmodium* as depicted in Fig. 3a, b.

Parasite at ring stage was used for compound concentration response assay with parasitaemia of 1% at 5% haematocrit. All chalcones, chloroquine, quinine and artemisinin were tested for anti-malarial activity on both strains by looking at percentage inhibition in schizont maturation following WHO Mark III protocol [36] in serially diluted range (6.25–0.09 μg/mL except artemisinin used in 6.25–0.09 ng/mL) of each drug concentration, Fig. 3c, d. The IC_50_ and IC_90_ values of all compounds were determined and the resistance index between the two sensitive and resistant strains was calculated (Table 2). The IC_50_ values acquired for all chalcones were in the range of 0.10–0.40 μg/mL for MRC-2 and 0.14–0.55 μg/mL for RKL-9. The chalcones 7 and 2 showed maximum potency with IC_50_ values of 0.11 and 0.13 μg/mL for MRC-2, and 0.18 and 0.14 μg/mL for RKL-9. The percentage inhibition in schizont maturation was also calculated after incubation of ring stage *P. falciparum* till 24 h at the same range of drug concentrations (Table 3).

**Table 2:** In vitro anti-malarial activity of the chalcones on *P. falciparum* chloroquine^S^ and *P. falciparum* chloroquine^R^ strains, their HeLa cell cytotoxicity and resistance (RI) and selectivity indices (SI)

Compounds/drugs code	*P. falciparum* Chloroquine^s^ Strain (MRC-2) IC_50_ (µg/mL)	*P. falciparum* Chloroquine^s^ Strain (MRC-2) IC_90_ (µg/mL)	*P. falciparum* Chloroquine^R^ Strain (RKL-9) IC_50_ (µg/mL)	*P. falciparum* Chloroquine^R^ Strain (RKL-9) IC_90_ (µg/mL)	Resistance Index (RI) IC_50_ (RKL-9)/IC_50_ (MRC-2)	HeLa Cell CC_50_	Selective index (SI) *P.falciparum* chloroquine^s^ (MRC-2)	Selective index (SI) *P.falciparum* chloroquine^R^ (RKL-9)
1	0.34	5.54	0.23	1.42	0.68	4.36	12.82	18.96
2	0.13	0.51	0.14	0.36	1.08	1.06	8.15	7.57
3	0.17	0.75	0.29	1.15	1.71	7.79	45.82	26.86
4	0.15	0.40	0.51	2.26	3.40	0.84	5.60	1.65
5	0.16	1.08	0.23	0.92	1.43	8.45	52.81	36.74
6	0.35	5.23	0.19	0.98	0.54	1.66	4.74	8.74
7	0.11	0.46	0.18	0.67	1.64	15.31	139.18	85.05
8	0.29	1.01	0.26	5.31	0.90	2.20	7.59	8.46
9	0.25	0.96	0.21	0.95	0.84	1.65	6.60	7.85
10	0.20	0.89	0.19	0.81	0.95	1.88	9.40	9.90
CHL	0.17	1.14	–	–	–	31.04	182.58	–
QNN	–	–	0.25	1.59	–	30.31	–	121.24
ART (ng/mL)	–	–	0.15	0.15	–	49.11	–	327.4

Chloroquine^S^ = Chloroquine Sensitive and Chloroquine^R^ = Chloroquine Resistant

*CHL* chloroquine, *QNN* quinine hydrochloride, *ART* artemisinin

**Table 3:** Schizont maturation inhibition (%) and haemolysis of normal erythrocytes (%) with effect to the chalcones

Drugs/compound	% Schizont maturation inhibition ± SD (MRC-2) (Conc. = 6.25 μg/mL)	% Schizont maturation inhibition ± SD (RKL-9) (Conc. = 6.25 μg/mL)	% Hemolysis ± SD (Conc. = 12.5 μg/mL)
1	82.43 ± 20.51	49.66 ± 25.46	1.44 ± 0.005
2	71.76 ± 10.61	58.02 ± 21.21	0.86 ± 0.002
3	59.26 ± 4.95	63.71 ± 2.83	1.01 ± 0.003
4	47.48 ± 17.68	57.69 ± 7.78	0.94 ± 0.006
5	58.65 ± 7.78	40 ± 20.51	0.65 ± 0.001
6	51.05 ± 42.42	43.85 ± 33.23	1.15 ± 0.005
7	94.24 ± 2.21	85.82 ± 6.36	1.08 ± 0.001
8	75.17 ± 12.02	77.78 ± 1.41	0.83 ± 0.003
9	50.32 ± 33.23	48.87 ± 25.46	1.30 ± 0.009
10	64.44 ± 9.90	49.62 ± 19.79	1.51 ± 0.013
CHL	88.39 ± 0.71	–	0.86 ± 0.001
QNN	–	71.87 ± 16.26	2.16 ± 0.035
ART (ng/mL)	–	87.88 ± 2.12	1.94 ± 0.026

*CHL* chloroquine, *QNN* quinine hydrochloride, *ART* artemisinin

### Cytotoxicity assay and evaluation of selectivity index

Compound cytotoxicity performed on HeLa cell line showed 50% inhibitory cellular cytotoxicity at concentration range from 0.80 to 16.00 μg/mL. The results are summarized in Table 4. The calculated selectivity index shown in Table 2 was 139.18 for 7, 52.81 for 5 and 45.82 for 3 and others had < 15.00 on the chloroquine sensitive strain. Similarly, 7 had higher selectivity index (85.05) as compared to other derivatives on the chloroquine resistance strain.

**Table 4:** Cell viability of chalcones and standard compounds on HeLa cell line (%)

Compounds	% Cell viability ± SD (Conc. = 12.5 µg/mL)
1	39.80 ± 0.06
2	47.57 ± 0.11
3	40.29 ± 0.07
4	33.98 ± 0.08
5	50.24 ± 0.08
6	41.74 ± 0.03
7	51.21 ± 0.04
8	42.71 ± 0.06
9	55.09 ± 0.09
10	47.08 ± 0.10
CHL	58.00 ± 0.06
QNN	62,62 ± 0.06
ART (ng/mL)	58.92 ± 0.06

*CHL* chloroquine, *QNN* quinine hydrochloride, *ART* artemisinin

The percentage viability of HeLa cells at different concentrations (12.5–0.09 μg/mL) of all compounds including standard anti-malarials is depicted in Additional file 1: Figure S1. At the highest concentration of 12.50 μg/mL (Table 4), the percentage cell viability of 9, 7, and 5 was more than 50%, which was found to be satisfactory compared to chloroquine (58.00 ± 0.06) and quinine (62.62 ± 0.06).

### Effect on fresh erythrocytes (haemolysis)

Fresh erythrocytes treated with chalcones derivatives for 3 h at different concentrations in serial dilution (12.5–0.09 μg/mL) showed minimal percentages haemolysis below 5% (Table 3) when compared to the standard control triton X-100 (100% haemolysis).

### Effect on haemozoin production

**Fig. 4:**
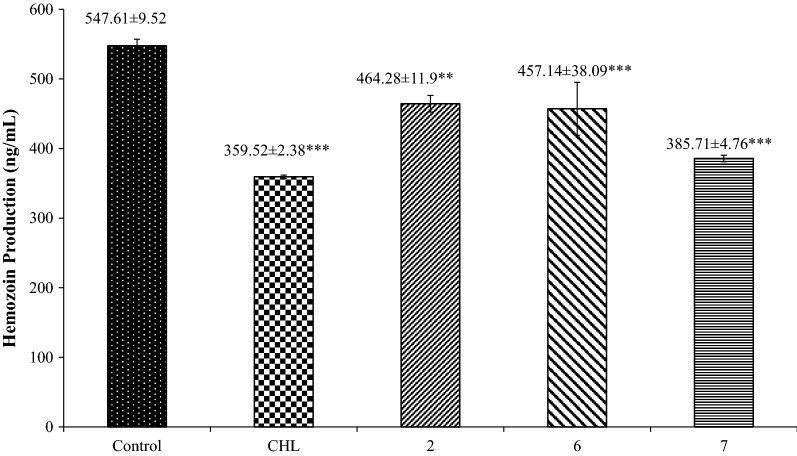
Amount of haemozoin production (ng/mL). n = The experiment was performed in triplicate. The data are represented as mean ± SD. Significant data are given as *p < 0.05; **p < 0.01;***p < 0.001

The quantity of haemozoin formed is directly related to the level of haemoglobin digestion. Data for the haemozoin production by the *Plasmodium* in the effect of chloroquine and the three most potent screened chalcone **2**, **6**, and **7** derivatives are represented in Fig. 4. The haemozoin production in non-treated infected erythrocytes was used as the positive control. The level of haemozoin production of the chalcone **7** (385.71 ± 4.76) was slightly higher than that of chloroquine (359.52 ± 2.38). Other chalcones also had lower level as compared to that of the control (547.61 ± 9.52).

## Discussion

Chloroquine and quinine retain anti-malarial efficacy for past several decades. Afterwhile, artemisinin-based combination therapy is the most recommended therapy to curb any malaria [37]. However, due to appearance of drug resistance and failure to achieve desired anti-malarial efficacy of existing drugs in several part of world [3] emphasizes the effort made by pharmaceutical companies and research organizations to search for new leads with high efficacy and minimal toxicity. Most anti-malarial drugs, such as chloroquine, quinine, mefloquine, halofantrine, pyrimethamine, sulfadoxine, sulfones, tetracyclines, act on the erythrocytic stage of parasite during the course of infection, which is the primary symptomatic phase of infection, thereby terminating the clinical attacks of malaria and addressing the constant threat of drug resistance [38]. Erythrocytic stages in culture of *P. falciparum* under in vitro conditions is practically feasible with easier manipulation step in the laboratory and found to be a major initial tool to screen schizontocidal compounds. Though this morphological microscopic method is cumbersome and labour intensive, it has been established because of its reproducibility and simplicity. It is also an inexpensive assay in comparison to the various other anti-malarial assays like [^3^H]-hypoxanthine incorporation assay, lactate dehydrogenase (pLDH) assay, Malaria SYBR Green I-based fluorescence (MSF) assay, double-site enzyme-linked lactate dehydrogenase enzyme immunodetection (DELI) assay, flow cytometric haemozoin detection assay, luciferase-based high-throughput screening (HTS) assay [39, 40], that can be set up in smaller laboratories. The evidence for the anti-malarial activity of chalcones from natural [16, 18, 41] and synthetic source is well documented [42–48].

In this study, 10 chalcones were analysed for anti-malarial activity and the results showed good activity against both chloroquine sensitive (MRC-2) strain (0.12–0.36 µg/mL) and chloroquine resistant (RKL-9) strain (0.15–0.52 µg/mL). The chalcones **7**, **2** and **6** showed maximum anti-malarial potency as the most potent of them, **7**, caused 94.24 ± 2.21% inhibition at concentration of 6.25 μg/mL (Table 3). In comparison, the chalcones with anti-malarial activity, described so far in the literature, have IC_50_ values between 1.1 and 12.3 µg/mL [11, 44, 47–49].

**Fig. 5:**
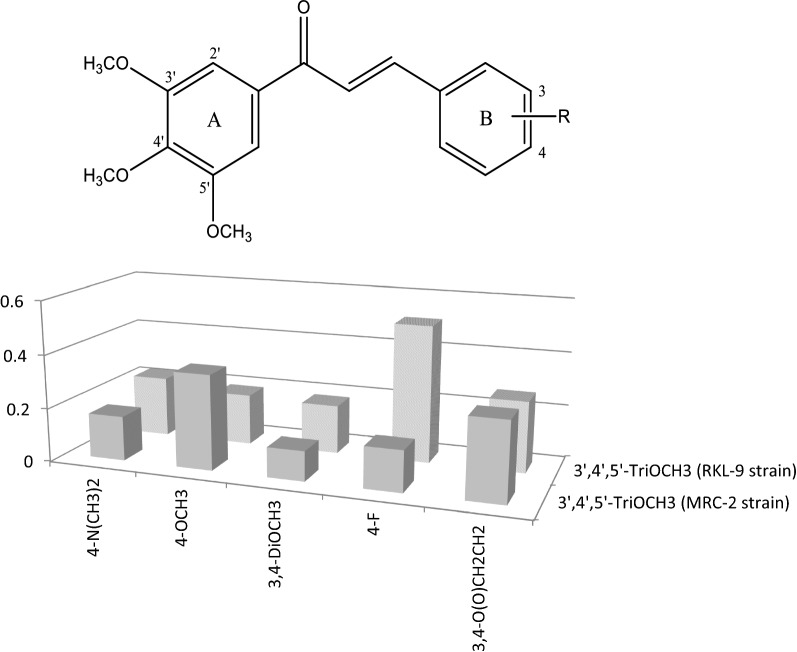
Influence of the substitution of the 3′,4′,5′-trimethoxychalcones on their anti-malarial activity

The anti-plasmodial activity of chalcones related to the position of methoxy groups on rings A and B. Concerning ring A, the most successful pattern was that of the 3′,4′,5′-trimethoxyphenyl motif (Fig. 5) shown by the chalcone **7**, which is a pharmacophore with diverse range of biological actions including anticancer, anti-invasive, antioxidant, and anti-inflammatory activities. Its effectivity against the MRC-2 strain decreased depending on the substituents in ring B, as followed: 3,4-dimethoxyphenyl-(3,4-diOCH_3_)>4-fluorophenyl-(4-F)>4-dimethylaminophenyl-(4-*N*(CH_3_)_2_)>3,4-methylenedioxyphenyl-(3,4-O(O)CH_2_CH_2_)>4-methoxyphenyl-(4-OCH_3_), while against the resistant strain, RKL-9, this order changed to: 3,4-diOCH_3_>4-OCH_3_ > 4-*N*(CH_3_)_2_>3,4-O(O)CH_2_CH_2_>4-F. This result shows that the presence of methylated hydroxyl and amino groups in ring B is more relevant to activity of the 3′,4′,5′-trimethoxychalcones against the chloroquine resistant strain, which might be useful for a future design of more potent chalcones with anti-malarial activity. However, exact relation between such substitutions patterns on ring B and anti-malarial activity is not known.

Meanwhile, one of the most active chalcones (**2**) possesses methoxy groups at C-2′ and C-5′ positions in ring A and at C-3 and C-4 in ring B, meaning that exploring anti-malarial activity of a larger series of 2′,5′-dimethoxy chalcones with various substituents in ring B is also worthy.

Further, to investigate cytotoxic effect of all these derivatives, results demonstrate very low cytotoxic activity of all derivatives. The chalcones 2, 6 and 7 produced minimal cytotoxicity. The selectivity index is defined as relative effectiveness of investigational compound in inhibiting cell proliferation as compared to inducing cell death. Therefore, it is preferable to have higher selective index that means maximal activity with least cellular toxicity [50]. The comparison of the SI values obtained for **7** and the reference compounds (chloroquine, artemisinin, and quinine) demonstrates good therapeutic effect of **7** and activity close to that of the reference drugs. The chalcone **7** has comparatively higher CC_50_ values (> 15.30 μg/mL) and good selective index (> 136.60) that defines optimum selective anti-malarial. Previously, Lim et al. [49], showed the most active chalcone in their study also having 3,4-diOCH_3_ substituents in ring B, but 2′-OH and 4′-OCH_3_ groups in ring A had prominent cytotoxicity towards FM3A cells, a model of the host, that has comparatively low EC_50_ values (> 3.3 μg/mL), indicating that the compound has non-selective anti-malarial activity. This shows that finding out the specific anti-malarial target is crucial for the design of chalcones with anti-malarial activity.

To evaluate the effect of all chalcones on normal erythrocytes, percentage haemolysis was measured. All the derivatives irrespective of the concentration range used in the study illustrate minimal haemolytic effect and did not shows any adverse events on erythrocytes at drug concentrations at which they eliminate the parasite which suggests that the anti-malarial effect of these chalcones were primarily not due to erythrocyte lysis.

Next to locate the chalcones, anti-malarial target, the study used to appraise the feasible inhibitory activity of the potent chalcones in haemozoin inhibition assay. The chalcones are supposed to interface and prohibit the *P. falciparum* cysteine protease (falcipain) action, a vital enzyme believed to be intricate in the haemoglobin digestion present inside the acidic food vacuole of the intra-erythrocytic parasite. Hindrance in haemoglobin digestion process is catastrophic for the *Plasmodium*. It is anticipated that malarial aspartic proteases (plasmepsin) and cysteine proteases (falcipain) mediate the haemoglobin digestion for releasing amino acids that are needed for intra-erythrocytic parasite multiplication and growth [51]. Also, these proteases form an interesting anti-malarial drug target [51]. Structure based analysis anticipate anti-malarial chalcones restriction on trophozoite cysteine protease as the probable mode of action [23]. The results showed significant reduction in the production of haemozoin when infected erythrocytes were treated with chloroquine and three other potent derivatives (**2**, **6**, and **7**), compared to untreated infected erythrocytes. This also suggests the similar mechanism of anti-malarial action of chalcones as the chloroquine does. Similar results were shown in the previous studies where different chalcone derivatives showed hindrance of plasmodial haemozoin formation in culture suggesting that these chalcones act on haemozoin formation pathways [52–54]. However, few studies reported that some do not interfere with haemozoin formation [55, 56]. This variation is mostly due to substitution on the ring A or B of the chalcones.

## Conclusion

Chalcones offer a very large repository of bioactive compounds with diverse molecular targets. Chalcones with even minor structural changes can result in targeting distinct cellular processes. The present in vitro study clearly indicates that finding the particular anti-malarial target is crucial for the design of potent chalcones. All chalcones here demonstrated potent anti-malarial activity in schizont maturation assay, with **7** having the highest potency (IC_50_ of 0.11 µg/mL) in contrast to licochalcone (1.43 µg/mL). Also, the inhibition in haemozoin production by these compounds suggests similar mechanism of action with chloroquine. However, extensive in vivo study is needed to confirm efficacy of these derivatives under influence of various physiological mechanism under-going inside animal models.

## Supplementary information

**Additional file 1: Figure S1.** Cell viability (%) of chalcones and standard compound at different concentrations; CHL-Chloroquine; QNN-Quinine hydrochloride; ART-Artemisinin.

## Data Availability

All data generated or analysed during this study are included in this published article and its additional files.

## Abbreviations

ANOVA: analysis of variance
CC_50_: cytotoxicity concentration 50%
DELI assay: double-site enzyme-linked lactate dehydrogenase enzyme immunodetection assay
DMEM: Dulbecco Modified Eagle Medium
DMSO: dimethyl sulfoxide
EC_50_: half maximal effective concentration
HEPES buffer: *N*-(2-hydroxyethyl)piperazine-*N*′-(2-ethanesulfonic acid) buffer
HTS assay: high-throughput screening assay
IC_50_: concentration for 50% inhibition
MSF assay: malaria SYBR green I-based fluorescence assay
MTT: 3-(4,5-dimethylthiazol-2-yl)-2,5-diphenyltetrazolium bromide
NAOH: sodium hydroxide
NCCS: National Centre for Cell Science
NIMR: National Institute of Malaria Research
PBS: phosphate-buffered saline
pLDH assay: parasite lactate dehydrogenase assay
RI: resistance index
RPMI media: Roswell Park Memorial Institute media
SD: standard deviation
SDS: sodium dodecyl sulfate
SI: selectivity index
SPSS: Statistical Package for the Social Sciences
WHO: World Health Organization
