# Supplementary figures and images for: In vitro anti-malarial efficacy of chalcones: cytotoxicity profile, mechanism of action and their effect on erythrocytes
Source: Malar J. 2019 Dec 16;18:421. doi: 10.1186/s12936-019-3060-z (PMC6916019; doi:10.1186/s12936-019-3060-z)

## Slide 1
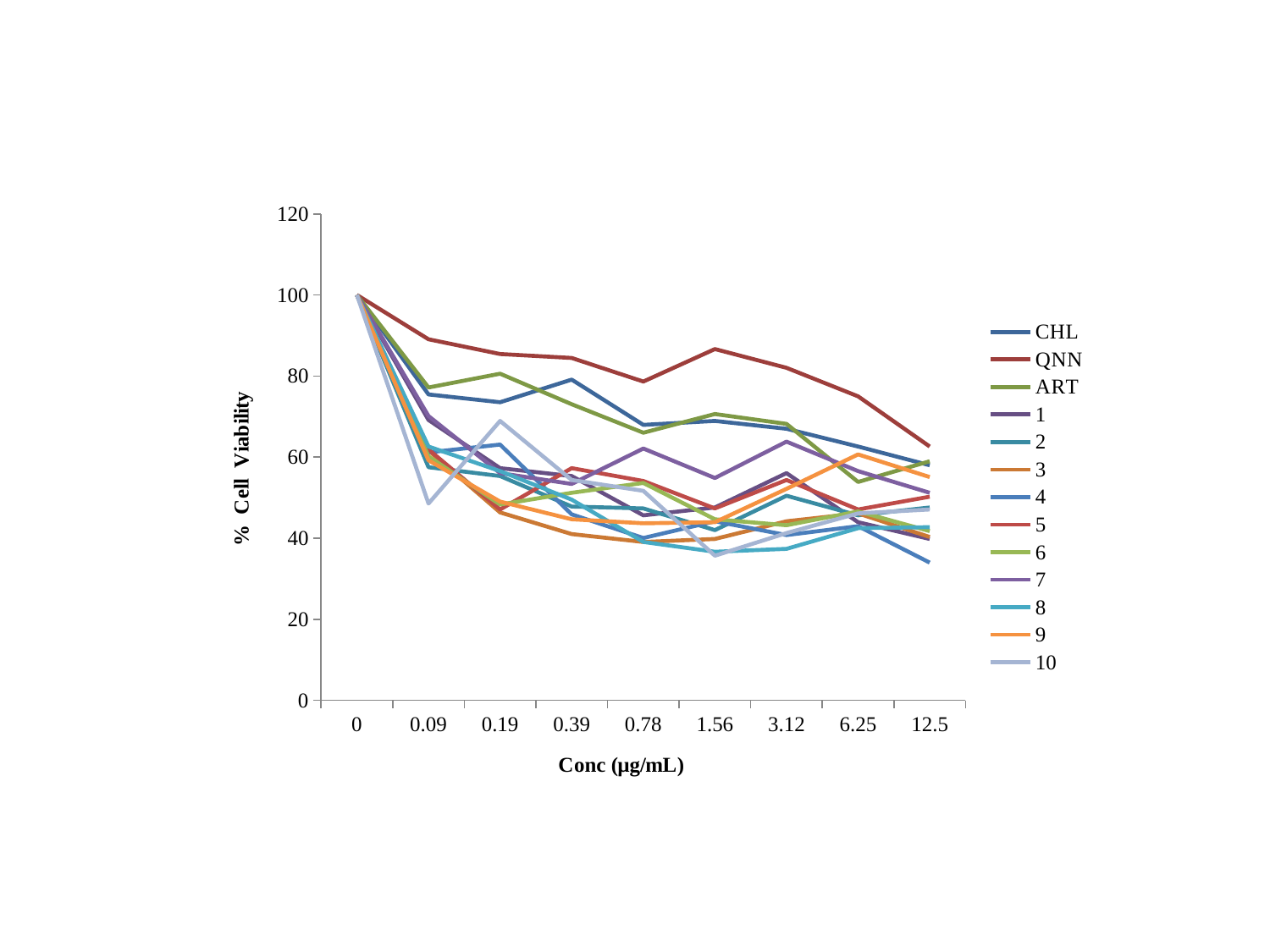

### Chart
| Category | CHL | QNN | ART | 1 | 2 | 3 | 4 | 5 | 6 | 7 | 8 | 9 | 10 |
|---|---|---|---|---|---|---|---|---|---|---|---|---|---|
| 0 | 100.0 | 100.0 | 100.0 | 100.0 | 100.0 | 100.0 | 100.0 | 100.0 | 100.0 | 100.0 | 100.0 | 100.0 | 100.0 |
| 0.09 | 75.46 | 89.07 | 77.18 | 69.17 | 57.52 | 61.65 | 61.16 | 61.89 | 60.19 | 70.14 | 62.62 | 59.22 | 48.54 |
| 0.19 | 73.54 | 85.43 | 80.58 | 57.28 | 55.33 | 46.35 | 63.1 | 47.08 | 48.3 | 56.06 | 56.55 | 49.02 | 68.93 |
| 0.39 | 79.12 | 84.46 | 73.05 | 55.33 | 47.81 | 41.01 | 45.87 | 57.28 | 51.21 | 53.39 | 49.51 | 44.66 | 54.36 |
| 0.78 | 67.96 | 78.64 | 66.02 | 45.63 | 47.33 | 39.07 | 40.04 | 54.12 | 53.64 | 62.13 | 39.07 | 43.68 | 51.69 |
| 1.56 | 68.93 | 86.65 | 70.63 | 47.57 | 41.99 | 39.8 | 44.17 | 47.33 | 44.66 | 54.85 | 36.65 | 43.93 | 35.67 |
| 3.12 | 66.99 | 82.03 | 68.2 | 56.06 | 50.48 | 44.17 | 40.77 | 54.36 | 43.2 | 63.83 | 37.37 | 52.18 | 41.26 |
| 6.25 | 62.62 | 75.00000000000001 | 53.88 | 43.93 | 45.63 | 46.11 | 42.96 | 47.08 | 46.6 | 56.55 | 42.47 | 60.67 | 46.11 |
| 12.5 | 58.0 | 62.62 | 58.98 | 39.8 | 47.57 | 40.29 | 33.98 | 50.24 | 41.74 | 51.21 | 42.71 | 55.09 | 47.08 |

Supplement: Supplementary file 1 — Additional file 1: Figure S1. Cell viability (%) of chalcones and standard compound at different concentrations; CHL-Chloroquine; QNN-Quinine hydrochloride; ART-Artemisinin. [file 12936_2019_3060_MOESM1_ESM.pptx]
